# Disparity of Age in Marriage

**Published:** 1854-11

**Authors:** 


Disparity of age in Marriage.—Mahomet's first wife, Kadyah, was at least 40, when he at the age of 25 married her. Shakspeare's Ann Hathaway was seven years his senior. Dr. Johnson's was literally double his age. The wife of Lord Herbert Cherbury six or seven years older than her lord. Sir Thomas Moore's wife was also seven years older than her husband. Howard, the philanthropist, at the age of 25 married a first wife, who was then 52. Mrs. Rowe, the authoress, was 15 years older than Mr. Rowe. Rapel, the German DeStael, was about as much older. The Countess D'Osili (Miss Fuller) was nearly ten years her husband's senior. Jenny Lind, too, is said to be eight or ten years older than Herr Goldsmidt.

